# Bilateral Cavernous Sinus Thrombosis Secondary to Dental Infection-Induced Sinusitis: A Case Report

**DOI:** 10.7759/cureus.105560

**Published:** 2026-03-20

**Authors:** Mark Genkin, Tony Joseph, Mary Budin, Jana Ghulmiyyah, Rena Kravitz

**Affiliations:** 1 General Dentistry Department, New York University College of Dentistry, New York, USA; 2 Chemistry Department, Brooklyn College, New York, USA; 3 Dental Department, Maimonides Medical Center, New York, USA; 4 Otolaryngology Department, Maimonides Medical Center, New York, USA

**Keywords:** caries, cavernous sinus thrombosis, infection, otomastoiditis, pediatric, sinusitis

## Abstract

Cavernous sinus thrombosis (CST) is a rare but severe condition that often arises as a complication of head and facial infections. Sinusitis, particularly involving the sphenoid or ethmoid sinuses, is a common precursor. A five-year-old boy presented to the pediatric intensive care unit with fever, ear pain, and eyelid swelling. Magnetic resonance imaging revealed rare bilateral CST with significant intracranial arterial narrowing. Cultures from the ear grew *Fusobacterium necrophorum*. Subsequent dental examination identified multiple untreated carious lesions, pulpal necrosis, and abscesses. Comprehensive dental rehabilitation, including extractions, pulpotomies, and stainless-steel crowns, was performed under general anesthesia in conjunction with otolaryngologic management. Following treatment, the CST resolved. This case illustrates a potential pathophysiological cascade where untreated dental caries contributed to sinusitis and otomastoiditis, ultimately leading to CST. The absence of valves in the facial and dural venous systems facilitates retrograde spread of infection, underscoring the importance of oral health in preventing severe systemic complications. While a direct microbial link was not confirmed, we postulate that the infection correlated with poor dentition, leading to sinusitis and subsequent CST. Bilateral CST is an uncommon but life-threatening condition that may originate from odontogenic sources. Prompt multidisciplinary evaluation, including dental assessment, is critical in achieving favorable outcomes in pediatric patients.

## Introduction

Cavernous sinus thrombosis (CST) is a rare but severe complication arising from infections of the face, ears, or paranasal sinuses, affecting the cavernous sinus, a major venous structure located at the base of the brain. CST is characterized by blood clot formation within the sinus, leading to neurological deficits, vision loss, proptosis, and other serious complications [1].

The absence of valves in the facial and dural venous systems permits retrograde blood flow, facilitating the spread of infection and subsequent thrombosis [2]. Although sinusitis and otomastoiditis are common risk factors for CST, other sources, such as dental infections, are less commonly recognized. Among microbial causes, *Staphylococcus aureus* is the most frequent pathogen, accounting for approximately 67% of cases; however, *Fusobacterium necrophorum*, an anaerobic gram-negative rod that is a resident of the oral and upper respiratory tract flora, is implicated in only 5% of cases [3].

Here, we present a case of rare bilateral CST complicated by otomastoiditis and sinusitis in a pediatric patient with no underlying autoimmune disease, where poor dentition and untreated dental caries were suspected contributing factors. This case highlights the importance of considering dental sources when diagnosing and managing CST, particularly when concurrent sinus and ear infections are present.

This case report has been previously presented at the Greater New York Dental Meeting on December 1st 2024.

## Case presentation

**Figure 1:**
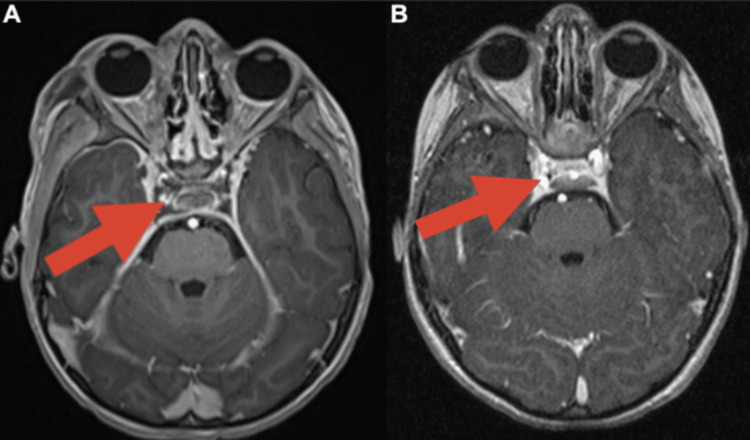
MRI comparison of a cavernous sinus thrombosis patient and a healthy patient. A: T1 post-contrast (magnetization-prepared rapid gradient-echo, MPRAGE) axial MRI at the level of the cavernous sinus demonstrates non-opacification of the cavernous sinus following the administration of intravenous contrast. B: For comparison, in a healthy patient, there is contrast opacification demonstrated in the cavernous sinus on a similar sequence post-contrast MRI.

A five-year-old male was admitted to the Pediatric Intensive Care Unit of Maimonides Medical Center with a fever, right ear pain, drainage, and right eye ptosis, common signs of CST in the setting of otogenic infection. Prior to this presentation, the patient had dental pain and extensive untreated caries, as well as pulpal necrosis, which was found upon dental examination. Laboratory tests revealed leukocytosis with predominance of neutrophils (left shift), elevated inflammatory markers, and electrolyte abnormalities. Cerebrospinal fluid analysis indicated mild pleocytosis of 43 white blood cells with a left shift. Magnetic resonance imaging (MRI) of the brain and orbits with and without contrast (Figure 1A) revealed bilateral CST and narrowing of the left intracranial internal carotid arteries as compared to a healthy control (Figure 1B). A filling defect of the left jugular bulb severely narrowed the lumen, suggesting a thrombus. Bilateral tympanomastoid and right external auditory canal opacification with internal diffusion restriction caused concern for acute otomastoiditis, which was later diagnosed. Markedly narrowed bilateral intracranial internal carotid arteries, most notably in the distal petrous and cavernous segments, with reconstitution, confirmed a CST diagnosis.

Ear cultures showed gram-negative rods of *Fusobacterium necrophorum*, and thus, vancomycin initially given was discontinued. The following morning, during bilateral myringotomy tube placement, the right external auditory canal, which had filled with thick, purulent secretions, was suctioned. The tympanic membrane was erythematous, bulging, and markedly thin.

**Figure 2:**
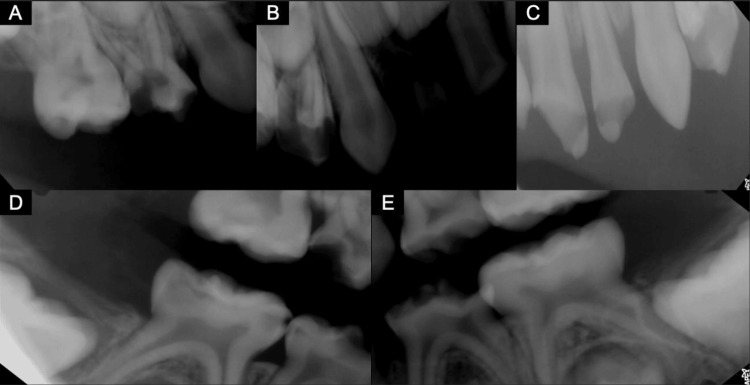
Dental radiographic imaging findings. The following numbered teeth correspond to the Fédération Dentaire Internationale (FDI) tooth numbering system for primary dentition. The first digit corresponds to the quadrant of the mouth, and the second digit corresponds to the tooth's position within that quadrant, numbered from the midline posteriorly [4]. A: Teeth 54 and 55 received stainless-steel crowns, and tooth 52 received a pulpotomy in the maxillary right quadrant. B: Teeth 51 and 52 in the maxillary right quadrant were extracted. C: Teeth 61 and 62 were extracted, and tooth 64 received a stainless-steel crown in the maxillary left quadrant. D: Teeth 81 and 82 received stainless-steel crowns and pulpotomies in the mandibular right quadrant. E: Teeth 71 and 72 received stainless-steel crowns, and tooth 74 received a pulpotomy in the mandibular left quadrant.

An anterior-inferior myringotomy was performed, and thick mucus secretions in the lumen were suctioned. Ofloxacin drops were then added to stop further bacterial infection in the area. The patient was hospitalized two times for the same infection before the pediatric dental team was consulted for a possible dental origin. Radiographic imaging (Figure 2) revealed multiple cavities, deep decay, periodontitis, and abscesses. Extractions, pulpotomies, and stainless-steel crowns were performed under general anesthesia to minimize infection and eliminate decay, as documented by the Fédération Dentaire Internationale (FDI) system in Figure 2 [4]. Shortly after the oral infection was treated, CST resolved.

## Discussion

CST is a life-threatening condition in the cavernous sinus region, most frequently caused by septic thrombophlebitis. The mechanism of spread is caused by retrograde venous flow through the pterygoid and facial valveless systems, which allows infection to spread from the maxilla to the cavernous sinus. This disorder is known to be caused by superficial cutaneous infections, dental disease with periapical abscess formation, or sinusitis. Although sinusitis and facial infections are more common causes, dental infection is still a prominent cause in some cases. Here, we present a rare pediatric case of bilateral CST likely caused by sinusitis following untreated dental caries and infection.

Due to the untreated caries and pulpal necrosis in our patient, which is referenced in the radiographs in Figure 2, sinusitis may have arisen through oral bacterial infection. Tympanomastoid and sphenoid sinus opacification were also seen on the MRI in Figure 1A, indicating sinusitis. The sinusitis potentially led to retrograde venous spread through the pterygoid plexus to the cavernous sinus, which would lead to thrombosis. Given the symptoms found in the patient, including fever, right ear pain, orbital swelling, and ptosis, CST was further confirmed as the diagnosis. MRI displayed in Figure 1A further showed bilateral CST with severe narrowing of bilateral internal carotid arteries and opacification of tympanomastoid air cells and sinuses. The inflammatory mucosal thickening throughout the paranasal sinuses (most notably in the sphenoid sinuses) and right eye ptosis may be a result of increased venous pressure. Further infections like meningitis result from septic CST through a similar retrograde mechanism [5].

In our patient, *F. necrophorum* was found in ear fluid culture, but its presence in oral abscess culture is unknown. However, it is important to note the findings of other studies where *F. necrophorum *found within abscesses was found to have a link to sinusitis, which then led to CST [2,3,6]. Also, culture-negative CST is also common in some studies due to fastidious anaerobes like *F. necrophorum*.

In order to initially treat the thrombosis, a myringotomy was performed with subsequent ear lumen suctioning and tympanostomy tubes placed, as well as antibiotics given. The pediatric dental department also performed a comprehensive dental rehabilitation with extractions, pulpotomies, and crowns placed under general anesthesia. The CST resolved after dental and otolaryngologic intervention.

This case is distinguishable in that it involves the simultaneous presence of rare bilateral CST and acute otomastoiditis in an otherwise healthy patient with no known history of immunodeficiencies. In this case, the concurrent presence of CST, otomastoiditis, and sinusitis raises concern for a shared origin, potentially initiated by poor oral health and complicated by spread to the sinuses and middle ear. A connection between dental decay, sinusitis, and CST has been identified previously [6-10], indicating possible dental origins of CST.

## Conclusions

This case highlights a rare presentation of bilateral CST potentially associated with sinusitis and otomastoiditis in a pediatric patient with no history of immune diseases. The isolation of *Fusobacterium necrophorum* from the ear further underscores the importance of identifying different sources of infection. Given the presence of significant dental decay and abscesses, alongside the valveless venous pathways facilitating retrograde infection spread, a dental source of infection is possible. This case emphasizes the need for early recognition of CST and prompt multidisciplinary intervention, including dental, otolaryngologic, and radiologic evaluation. Additionally, it highlights the importance of maintaining oral health to prevent life-threatening systemic complications. Ongoing research is essential to further clarify the pathogenesis of sinusitis-driven CST originating from dental infections and the role of pathogens such as *F. necrophorum*.
